# Oil Sorption Capacity of Recycled Polyurethane Foams and Their Mechanically Milled Powders

**DOI:** 10.3390/ma19010166

**Published:** 2026-01-02

**Authors:** Pierluigi Cossari, Daniela Caschera, Paolo Plescia

**Affiliations:** 1 Department of Physics, Institute of Nanotechnology CNR-NANOTEC, Sapienza University, 00185 Roma, Italy; 2 Institute for the Study of Nanostructured Materials, Lab Surface, ISMN-CNR, Strada Provinciale 35d/9, Montelibretti, 00010 Roma, Italy; 3 Institute of Environmental Geology and Geoengineering, CNR-IGAG, Strada Provinciale 35d/9, Montelibretti, 00010 Roma, Italy; ilplescia@gmail.com

**Keywords:** oil sorption, recycled polyurethane foams, mechanical grinding, plastic waste materials

## Abstract

Polyurethane (PU) is widely recognized for its efficient oil sorption properties. However, this capacity is highly dependent on its intrinsic chemical composition and morphological structure, which can be altered by mechanical or chemical treatments commonly applied before using it as a sorbent. In this study, we present a comprehensive investigation of the oil sorption behavior of both soft and rigid PU foams, and their blade-milled ground (BMG) counterparts obtained by mechanical treatment of several recycled PU-based products, including seats, mattresses, side panels of cars, packaging components, and insulating panels of refrigerators and freezers. We found that blade milling the soft PU foams leads to a significant reduction in oil sorption capacity proportional to the extent of grinding. Pristine soft PU foams and BMG-PUs with intermediate particle size (−250 μm–1 mm) exhibited the highest oil uptake (20–30 g/g), whereas the finest fraction (5 μm–250 μm) showed a lower capacity (3–7 g/g). In contrast, rigid PU foams showed consistently low oil sorption (~5 g/g), with negligible differences between the original and ground materials. At the macroscopic level, optical and morphological analyses revealed the collapse of the 3D porous network and a reduction in surface area. On the microscopic scale, spectroscopic, structural, and thermal analyses confirmed phase separation and rearrangement of hard and soft segmented domains within the polymer matrix, suggesting a different mechanism for oil sorption in BMG-PU. Despite reduced performance compared to pristine foams, BMG-PU powders, especially those with intermediate dimensions and originating from soft PU foams, present a viable, low-cost, and sustainable alternative for oil sorption applications, including oil spill remediation, while offering an effective strategy for effective recycling of PU foam wastes.

## 1. Introduction

Polyurethane (PU) foams are a versatile class of polymeric materials, where a tunable cellular structure and a combination of soft and hard segments form soft and rigid polymers with different degrees of microphase-separated structures. Thanks to this unique microstructure, PU exhibits a wide range of physical and mechanical properties, making it suitable for various applications in construction, transportation, the household, the military, and many other fields [1,2,3,4,5]. For this versatility, PU has been recognized as the “fifth-largest plastics” [6] due to its widespread and diverse applications, encompassing a vast range of products from everyday items of daily use, such as shoes, clothes, sports equipment, car components, furniture, and mattresses. Its high versatility, combined with properties like durability, resilience, and flexibility, makes it a staple in many industries [7,8,9,10,11,12]. In this context, the increasing quantity of PU wastes, their very long lifetimes due to their high durability and resistance toward solvents and compounds, and their long persistence in the environment make PU waste recycling an urgent task. Therefore, the end-of-life recovery and recycling of PU are currently of extreme importance to avoid landfill confinement or incineration due to the toxicity of the combustion process. In addition, recycling strategies of PU are in line with the elimination of crude oils (i.e., fossil fuels), which are widely used for the synthesis of PU [13]. Four major methods are used for PU recycling and recovery: mechanical recycling, advanced chemical and thermochemical recycling, energy recovery, and product recycling [13,14]. However, each of these methods provides advantages and disadvantages, and the choice of the most suitable procedure of recycling should be considered on a case-by-case basis, based on the specific details of each situation and, in particular, taking into consideration the economic, logistical, and ecological aspects.

With regard to mechanical recycling, fragmentation of PU wastes by grinding PU into fine particles (powder) is considered one of the most effective methods [15]. PU wastes, derived from appliances, automobiles, bedding, carpet cushions, upholstered furniture, parts of old components of refrigerators, and other consumer products, can be processed as sawdust or ground into a fine powder. Grinding is most frequently carried out in tumbling mills such as ball and rod mills, utilizing loose grinding media, lifted by the rotation of a drum, to break the ores in various combinations of impact, attrition, and abrasion to produce the specified product. The resulting polymer microstructures can be re-used in several applications spanning from the fabrication of rigid components, such as motor and pump covers, by compression and thermal treatment (350 bar and 180 °C), to fillers for other resins and re-bonding with adhesives [16], as well as for oil sorption and oil spill cleanup [17].

In particular, in the field of oil sorption application, PU foams were employed as absorbents for oil spill treatments, showing an intrinsic high sorption capacity in absorbing organic solvents and oils [18,19,20,21,22,23,24,25,26,27,28]. In fact, thanks to their hydrophobic nature and their high affinity with different types of hydrocarbons, PU foams can selectively absorb an amount of oil up to 10 times higher than their original weight without sinking into the sea [29]. However, while PU foams have been extensively investigated for these applications, no other work has reported, to the best of our knowledge, on the specific investigation of the oil sorption capacity and absorbing mechanism of PU powder from ground PU wastes. Some studies have reported an increase or modification in the surface area resulting from the disruption of closed cells [30,31]; nevertheless, little is known about the internal arrangements that the polymeric matrix may possibly undergo upon mechanochemical activation, nor how mechanical treatment can induce structural and chemical modifications in PU structures and consequently affect their oil sorption capacity.

In this context, we aim to investigate the possible relation between changes in the structural and morphological properties of blade-milled ground (BMG) PU powders induced by mechanical treatment and their oil sorption capability. For this purpose, we took into consideration several types of PU foam and the corresponding BMG-PU powders with two different particle dimensions obtained by blade milling of waste PU-based products, including seats, mattresses, side panels of cars, packing components, and insulating boards for refrigerators and freezers. To investigate the effect of mechanical action, a complete structural and morphological characterization of the PU foam wastes, both soft and rigid, before and after the blade-milling process, was carried out. In particular, optical and morphological tests were used to analyze the macroscopic modifications such as the rupture of the PU cells into fragmented structures of different sizes and shapes, whereas structural rearrangement and microphase separation between the hard and soft domains were highlighted by combining spectroscopic techniques with morphological and thermal analysis.

The differences in oil sorption capacity between the PU foams and BMG-PU powders with intermediate particle size (250 μm–1 mm) and the finest fraction (5 μm–250 μm) were then analyzed, correlating structural and microphase changes with the increases in surface reactivity and contact area between oil and polymer structures. These absorbing BMG-PUs can thus represent an alternative solution for effective oil sorption and, eventually, for oil spill cleanup, making the management of PU waste materials simultaneously more sustainable and less expensive.

## 2. Experiment

PU foams were recovered from a waste collection center in Rome and at the Auto Breaker Pomili Srl (Monterotondo, Rome, Italy), which specializes in the disposal and recycling of waste materials from the dismantling of household refrigerators, insulating components for buildings, and vehicles. Specifically, in the case of refrigerators, to obtain the recycled PU materials, several starting treatments were carried out in the center prior to collecting and grinding, i.e., the initial disassembling of the electrical components, the depollution of hazardous fluids and refrigerants, the extraction of the compressor, and the shredding of the exterior shell and inner plastic unit of refrigerators. Then, the mixed plastics were processed in another plant, while the ferrous and non-ferrous metal fractions were recovered through a magnetic separator by an electric arc furnace and eddy current separator, respectively. Before magnetic separation, the heterogeneous material resulting from the shredding phase was subjected to air separation in a “zig-zag” separator unit, obtaining PU foams (>1 cm; 1 cm–7 cm) for about 21.7% of the total mass. Finally, the PU foams were treated by a milling process to achieve particle sizes below 10 mm (1 cm) and then briquetted and sent for incineration. In this study we collected pieces of PU foams obtained after zig-zag separation (>1 cm; 1 cm–7 cm).

For both PU originating from insulating components (refrigerators) and vehicles, PU foams with dimensions ranging between 1 cm and 5 cm were collected and ground in a blade mill. The laboratory knife mill Polymix PX-MFC 90 D (Kinematica AG, Malters, Switzerland) was used for the experiment. Three knife mills can operate at constant revolutions of 900 min^−1^ with the diameter of the chamber being 150 mm. Pieces of PU foams were put in the feeding chamber of the mill manually, and about 50 gr of PU powder was obtained after 5 min. To optimize the grinding process of soft PU with the aim of obtaining powder of a smaller size, the samples were immersed in a liquid nitrogen bath and then pulverized by blade milling. Alternatively, blade milling of rigid PU foams was performed on pre-cut PU foam pieces at ambient temperature. In terms of grinding yields, the rigid PU foam yielded approximately between 65 and 80% of material below 1 mm, with 20–35% falling into the finest fraction (<250 µm). On the other hand, in the case of soft PU foams, less than 10–15% reached the 500 µm–750 µm fraction, and most of the materials remained in the 750 µm–1 mm dimensional range. For this reason we treated the soft PU foams with liquid nitrogen prior to milling with the aim of increasing the fine-particle yield. Under identical milling and sieving conditions, the mass fraction passing 500 µm increased to 55%, while the fraction of particles below 250 µm increased to about 40% (see Table S1). All yields are averages of three independent runs. The particle size distributions of the BMG-PU range from 0.5 μm to 2000 μm and were determined for both dry (air) and wet (water) particles by laser diffraction using a Mastersizer 2000 equipped with a Hydro 2000 (Malvern Panalytical Ltd., Malvern, UK). A single measurement of size distribution consisted of 15 repetitions lasting for over 10 s, taken at millisecond intervals. Optical images of ground particles were obtained with a Malvern Morphology G3 apparatus (Malvern Panalytical Ltd., Malvern, UK) equipped with a Nikon CFI 60 optical unit and a CCD digital camera (Nikon, Tokyo, Japan). This system allowed us to obtain reliable information on the size and shape of PU particles from 0.5 μm to several millimeters, as it was able to analyze a statistically significant number of particles. Finally, three stainless sieves with 18, 60, and 3250 meshes were used to separate powder with particle dimensions of about 1 mm, 250 μm, and 5 μm. Each fraction was collected separately and weighed, and its yield was expressed as a percentage of the total mass input.

Scanning electron microscopy (SEM) images of the PU foams and BMG-PU materials were acquired using a GEMINI-Supra 40 from Carl Zeiss (Oberkoken, Germany) with acceleration voltages ranging from 1 to 15 kV. AFM images were performed in tapping mode using a Scanning Probe Microscope from Digital Instruments (Veeco, Santa Barbara, CA, USA) with Nanosensor TESP (tapping etched silicon probe) type single beam cantilevers. These cantilevers had a nominal length of ca. 125 µm with force constants in the range of 20–100 N/m and were used at oscillation frequencies in the range of 200 and 400 kHz. The cantilevers had a very small tip radius of 5–10 nm. All images were obtained in air and collected over a range of scan sizes from 500 nm to 10 µm, depending on the particles analyzed. The BMG-PU powder was directly inserted in the AFM sample holder using double-sided adhesive tape.

Solid-state NMR measurements were carried out with a Bruker Avance 400 spectrometer (Bruker, Ettlingen, Germany) containing a 5 mm inverse detection z-gradient probe. The 13C NMR spectra at 9000 Hz were performed at 25 °C using deuterated dimethyl sulfoxide (DMSO-d6) as a solvent. Signals of carbon atoms are given on the δ scale relative to the signals from DMSO-d6.

X-ray diffraction (XRD) patterns were collected using a Rigaku Rint 1200 (Rigaku, Tokyo, Japan) equipped with Cu Kα radiation. The diffracted X-rays were detected using a NaI(Tl) scintillation counter point detector coupled with a graphite monochromator positioned in the diffracted beam. FTIR spectra were obtained using a VARIAN 1000 FTIR Scimitar Series spectrometer (Varian Inc., Palo Alto, CA, USA). Samples were prepared by mixing PU materials with KBr at a ratio of 1:100 *w*/*w* and pressing the resulting mixtures into cylindrical wafers. Thermogravimetric analysis (TGA) and differential thermal analysis (DTA) were performed using TA Instruments SDT-Q600 equipment (New Castle, DE, USA) in a temperature range from 25 °C to 800 °C with a heating rate of 10 °C min^−1^.

The study of oil sorption was conducted on waste PU foams and two particle-size fractions deriving from the pre-treatment and grinding process. The tests were carried out three times, each to ensure accuracy and reproducibility on the basis of the procedures outlined in the international ASTM F726–17 standard method [32], which is used for evaluating the capability and performance of sorbents in removing crude oils and related spills.

The oil sorption capacity was assessed according to the following formula:

$$S_{s}=\frac{W_{f}-W_{i}}{W_{i}}$$

where S*_s_* is the oil sorption capacity; W*_f_* is the weight of the sorbent at the end of the oil test after 2 min dripping; and W_i_ is the initial weight of the dry sorbent.

Specifically, the oil sorption capacity represents the amount of sorbed oil normalized by the absorbent’s mass. In this work, a diesel fuel (Quaser, produced by Q8) with a density of 0.845 and a kinematic viscosity of 3.9 mm^2^/s was used for oil sorption tests. The laboratory equipment consists of a crystallizer, a steel mesh of 0.1 mm, and an analytical balance. The procedure for the sorption tests is detailed as follows: the PU sample was placed in the steel mesh basket (0.1 mm mesh) and kept in contact with the oil poured in the crystallizing dish (2.5 cm height) for 15 min. After this period the sample was removed from the crystallizer, and the soaked absorbent with the mesh basket was drained for 10 min, allowing the release of the excess oil which was not completely absorbed. The weight measurements of the samples were taken after 0.5, 1, 2, 3, 5, 10, 15, 20, 25, 30, 45, 60, and 300 min in order to accurately evaluate the sorption capacity and the tendency to release.

## 3. Results and Discussion

### 3.1. Recycle of Dismantled PU Foams and Mechanical Treatment by Blade-Milling Process: Analysis of Size, Morphology, and Chemico-Physical Properties

**Figure 1:**
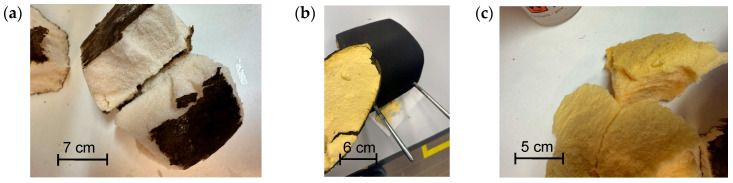
(**a**) Photographs of pieces of rigid PU foam from recycled insulating panels used in refrigerators, and (**b**,**c**) soft PU foams used for the cushioning of vehicle headrests.

Prior to mechanical treatment, rigid and soft PU foams were manually disassembled from end-of-life refrigerators and vehicle components, respectively, both at waste disposal centers and in the laboratory. As evident from Figure 1a–c, this straightforward procedure enabled the recovery of PU foams in medium to large sizes, which were subsequently cut into small pieces of 5–7 cm.

**Figure 2:**
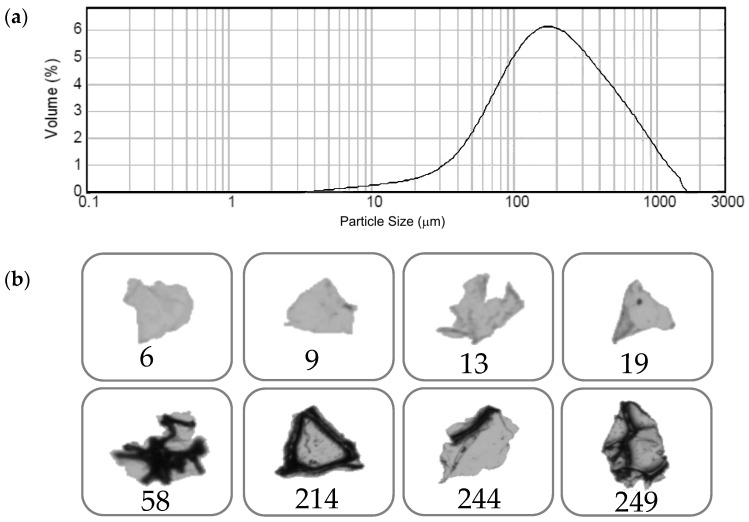
(**a**) Particle size distributions in the volume of BMG-PUs. (**b**) Typical shapes of BMG-PU particles obtained by blade-milling process of PU foam wastes. Different shapes and dimensions of BMG-PU particles within the ranges of 6–20 μm (**top**) and 50–250 μm (**bottom**). The numbers indicate the mean aerodynamic diameter in µm.

Each type of PU foam, derived from different end-of-life products, was milled separately in order to preserve the characteristics of the individual materials and for oil sorption tests. At the same time, mixed soft PU foams originating from various flexible products, including packing materials and vehicle components, such as car seats, steering wheels, and headrests, were collected and processed by blade milling. In both cases, BMG-PU powders with various particle sizes and size distributions were obtained, as clearly shown by laser diffraction analysis (Figure 2a). Data essentially indicated that the first mechanical fragmentation in the blade mill consisted of particles with diverse sizes, ranging between 2 μm and 1500–2000 μm (2 mm), with a broad, unimodal distribution centered at about 150 µm. Specifically, the volume of over 95% of the entire particle population in the particle size range was below 1000 μm, with a mean value of 133 μm. The analysis was also performed for wet particles (water) (see Figure S1), achieving similar results. Such a similarity in the size distributions for dry and wet BMG-PUs suggests the occurrence of well-dispersed systems with a low degree of agglomeration, associated with weak interactions between PU particles. Optical analysis of BMG-PUs, obtained by using the Morphologi G3 analyzer, clearly highlighted the presence of fragments and particles with different shapes and dimensions, ranging between 5–20 μm and 50–250 μm (Figure 2b). According to the *t*-test results, the measured BMG-PU particles were found to be statistically different in shape (*p* = 0.05). Particularly, great differences in shapes were observed for large particles, where it was still possible to recognize typical fragments of the bulk polymer, such as hard triangular and hexagonal shapes holding the polyhedral cells of the original PU foam. Fine particles with a dimension below 20 μm were mostly composed of the brighter soft phase of PU, whereas darker areas indicated lower light transmission of particles, which is associated with the hard domains of PU structures. Useful information about the relative distribution of soft and hard domains was also inferred by the light transmission profile of the BMG-PU material, which was measured in a statistically significant number of particles (n = 20,540), as shown in Figure S2. Transmission values were plotted against arbitrary light transmission units (a.l.t.u.), where 0 corresponds to a complete extinction of light and 260 to complete transmission. The profile was characterized by two maxima, with the more intense peak centered at about 140 a.l.t.u., and the other at 80 a.l.t.u. Both of them strongly overlapped, with two smaller peaks at 160 and 100 a.l.t.u., respectively. These results suggested that the majority of BMG-PU particles have the thin membranes of the soft phase (more transparent to light), whereas the fraction composed of the less transparent hard domains is significantly smaller.

### 3.2. Oil Sorption Capacity of PU Foams and Powder

For oil sorption measurements, each type of BMG-PU powder, including that obtained by milling mixed soft PU foams, was sieved and separated into two fractions with particle sizes ranging from 250 μm to 1 mm and from 5 μm to 250 μm. In terms of grinding yields, these differed notably between rigid and soft PU foams. In the first case, we achieved, at room temperature (RT), a higher amount of fine particles, probably due to the material’s brittle fracture behavior. On the other hand soft PU foams had a coarser fraction for their elastic recovery during cutting, thus requiring treatment with liquid nitrogen prior to blade milling (see Experiment Section for further details on grinding and sieving).

**Figure 3:**
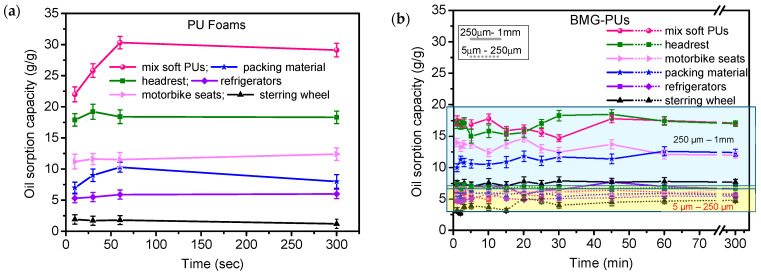
The oil sorption capacity (g/g) of the (**a**) PU foams, (**b**) BMG-PU powder with particle size ranging from 250 μm to 1 mm (solid lines) and from 5 μm to 250 μm (short dotted line).

PU foams derived from different types of industrial PU waste sources (e.g., automotive or packing materials) showed oil sorption capacities ranging from 10 g/g and 20 g/g (Figure 3a). In particular, it was observed that some samples, such as the steering wheel, exhibited very low efficiency, while the foams obtained by mixing soft PU components (pink line) demonstrated more effective sorption properties, reaching an oil sorption capacity of 30 g/g within 1 h. These values are in accordance with the literature, in which an efficiency of oil sorption in untreated PU foams is reported in the range of 20–40 g/g (see Table S2) [22,23,24,28]. The differences are attributed to the specific 3D hierarchical porous structure of PU foam, which allows water and organic solvents to flow easily into the material, promoting liquid penetration via capillary action.

Figure 3b shows the oil sorption capacity of the BMG-PU powders with two different particle sizes, ranging from 250 μm to 1 mm and 5 μm to 250 μm. Regardless of the primary source, BMG-PU particles with a smaller size (−5–250 µm) showed significantly lower oil sorption capacities compared to the corresponding PU foams with values ranging between 3 g/g and 8 g/g. Notably, soft mixed PU showed a decrease in its oil sorption capacity from 30 g/g to 6 g/g after milling (see Figure 3b and Figure S3). Improved performance was observed for the BMG-PU powder fraction with larger particles (250 µm–1 mm), where the oil sorption capacity ranged from 8 g/g to 20 g/g. Specifically, for the soft mixed PU, the oil sorption decreased from 30 g/g in foam to 20 g/g in the powder. Although reduced, this still represents a good result for a PU-based sorbent material. Nevertheless, these findings clearly indicate that the blade-milling procedure has a detrimental effect on the intrinsic oil sorption properties of PU foams. This is likely ascribable to the rupture of their original 3D open-cell structure, which is crucial for efficient oil uptake. At the same time, the variation in oil sorption performance among different BMG-PU powder size fractions is reasonable given the structural and morphological changes induced by the milling process. It is worth noting here that the management of the end of life for both PU foams and PU powder is essentially based on controlled incineration treatments with energy recovery. This allows us to dispose of oil–PU powder or PU foams under regulated environmental controls and with a positive energy gain, which is higher than that of neat PU. Another alternative strategy is oil recovery by mechanical squeezing. In this case the absorbed oil can be partially removed. However, unlike in PU foams, this route has limited applicability in PU powder since PU powder loses its mechanical integrity and foam structure and its consequent sorption reversibility and purity, which are typical of the foams and needed for effective oil removal.

Hereafter, to better elucidate the correlation between the observed changes in oil sorption capacity and the milling treatment, a comprehensive characterization of both the PU foams and resulting BMG-PU powders has been carried out, as presented in the following sections.

### 3.3. Analysis of Structural, Chemico-Physical Changes Induced by Mechanical Treatment

**Figure 4:**
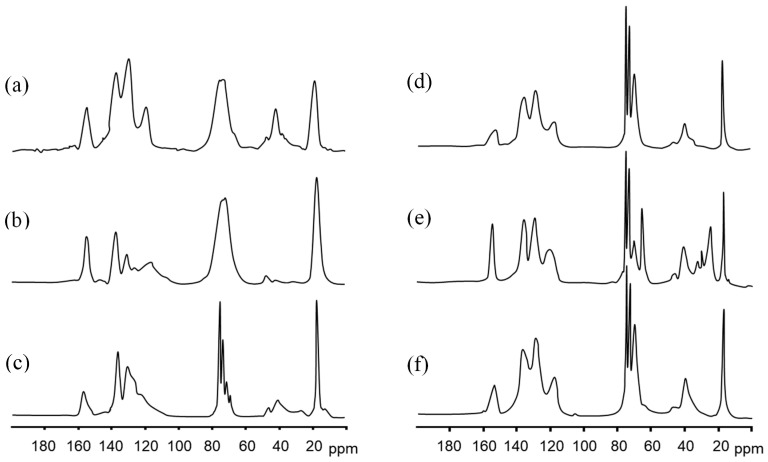
^13^C solid-state NMR spectra of recycled PU foams**:** (**a**) mixed soft PUs, (**b**) insulating panels for refrigerators, (**c**) packing materials, (**d**) motorbike car seats, (**e**) steering wheel, (**f**) car headrest.

Solid-state ^13^C NMR analysis of recycled PU foams:

PU foams originating from the dismantling of refrigerators and vehicle components were analyzed by ^13^C solid-state NMR spectroscopy in order to identify the chemical structure of each specific PU foam composition. It is worth mentioning here that PU foams are prepared by the polymerization reaction between a polyol and a polyisocyanate, forming urethane bonds. This process typically requires a catalyst or UV radiation. The resulting foams, either open-cell or closed-cell, are usually produced with one or more blowing agents added during the polymerization step [6]. Open-cell foams are characterized by ruptured lamellae between adjacent cells, resulting in interconnected porosity. In contrast, closed-cell foams retain intact lamellae, forming isolated gas-filled cells. PU foams are typically formed from randomly segmented copolymers consisting of soft segments and more rigid, urethane-rich hard segments. Their unusual elastomeric properties arise from the thermodynamic incompatibility between the soft and hard segments of the copolymer, leading to microphase separation, which in turn is governed by thermodynamic factors and the ability of the hard segments to pack efficiently and form hydrogen bonds [33,34]. The degree of phase separation is also affected by the chemical structure of PU chains, type of polyols, polarity of the structural fragments, and size and molecular weight of hard and soft segments [35,36]. Figure 4 shows the spectra of PU foams with the typical peaks assigned to polyols, including polypropylene oxide (PO) (–CH_3_; CH(CH_3_)–O; CH_2_–O– 12, 73 and 75 ppm, respectively), and aromatic isocyanates, methylene diphenyl diisocyanate (MDI) and 2,4 tolylene diisocyanate (TDI) (CH_3_–Ar 18, C arom. 121 and 126, aromatic ring–CH_3_ 136 ppm), as well as the urethane bond (–NH–Ar, –CO–NH–130 and 155 ppm) [37]. In particular, mixed soft PU foams (Figure 4a) and those originating from the recycling of packaging materials, motorbike seats, and car headrests (Figure 4d–f, respectively) exhibited peaks associated with MDI, PO, and PO end-functionalized with hydroxyl groups like ethylene oxide (EO) (PO-based polyether polyols, PEO). On the other hand, insulating panels used in refrigerators exhibited peaks associated with TDI and PO-PEO (Figure 4b,c), whereas PU foam from steering wheels showed a more complex structure made of a mixture of MDI and a modified isocyanate, polyether polyols made of low molecular weight PO-PEO, and additives. It is worth noting that rigid PUs are typically made from low molecular weight polyols (a few hundred), whereas soft PUs are composed of high molecular weight polyols [28].

^13^C solid-state NMR of mixed soft PU foams with higher absorption capacity (see the pink curve in Figure 3a) was also compared with that of the corresponding mixed soft BMG-PU powder, i.e., the PU ground powder with an intermediate size fraction (250 µm–1 mm), in order to evaluate potential modifications in chemical structure upon mechanical activation. This powder is light brown; had a bulk density of 0.16 g cm^−3^; an average content of water of 4% *w*/*w*; and exhibited a melting temperature of 220 °C. The amount of ash produced at 600 °C was 27% *w*/*w* (see Table S3).

**Figure 5:**
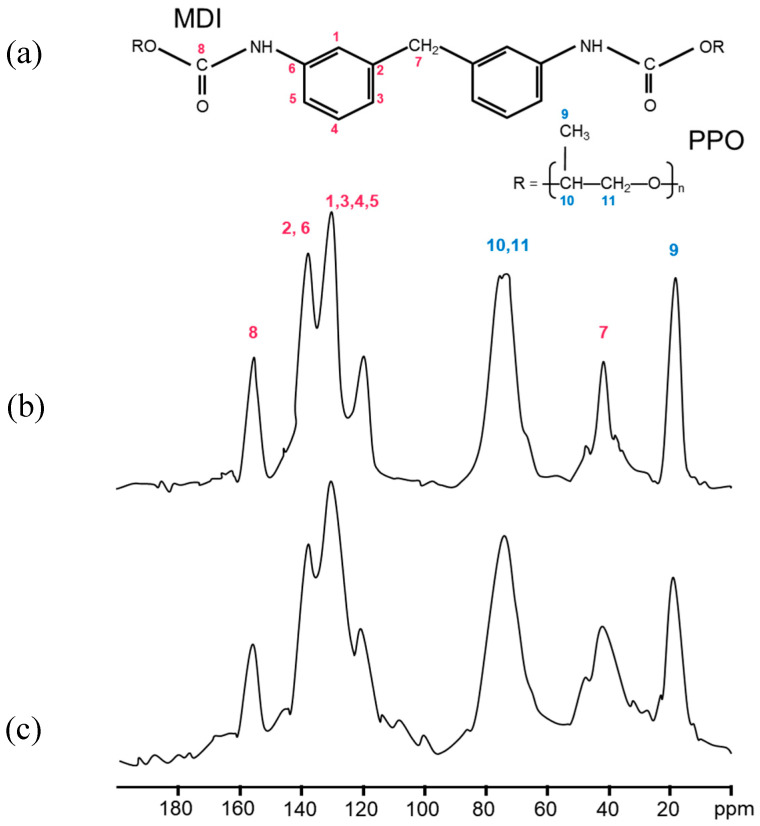
(**a**) Chemical structure and related ^13^C solid-state NMR signals of MDI and PPO. (**b**) ^13^C solid-state NMR spectra of mixed soft PU foams, and (**c**) the corresponding mixed soft BMG-PU with intermediate size fraction (250 µm–1 mm).

By comparing the NMR spectra (Figure 5), we did not observe any changes in the chemical shift positions except for a slight broadening of the signals. This suggested that the grinding process introduces structural disorder, enhances surface effects, and eventually reduces chain mobility near the fracture sites, whereas the chemical structure remains largely preserved. Indeed, as reported in the literature, decreased mobility leads to enhanced dipolar interactions and shortened transverse relaxation times, resulting in broader NMR lines.

### 3.4. Morphological, Structural, and Chemico-Physical Changes Induced by the Grinding Process

Hereafter, the morphological, structural, and chemico-physical changes induced by the grinding process were investigated, focusing on the more absorbing PU foams, i.e., soft mixed PU foams, and the corresponding BMG-PU particles with intermediate size fractions (−250 µm–1 mm).

**Figure 6:**
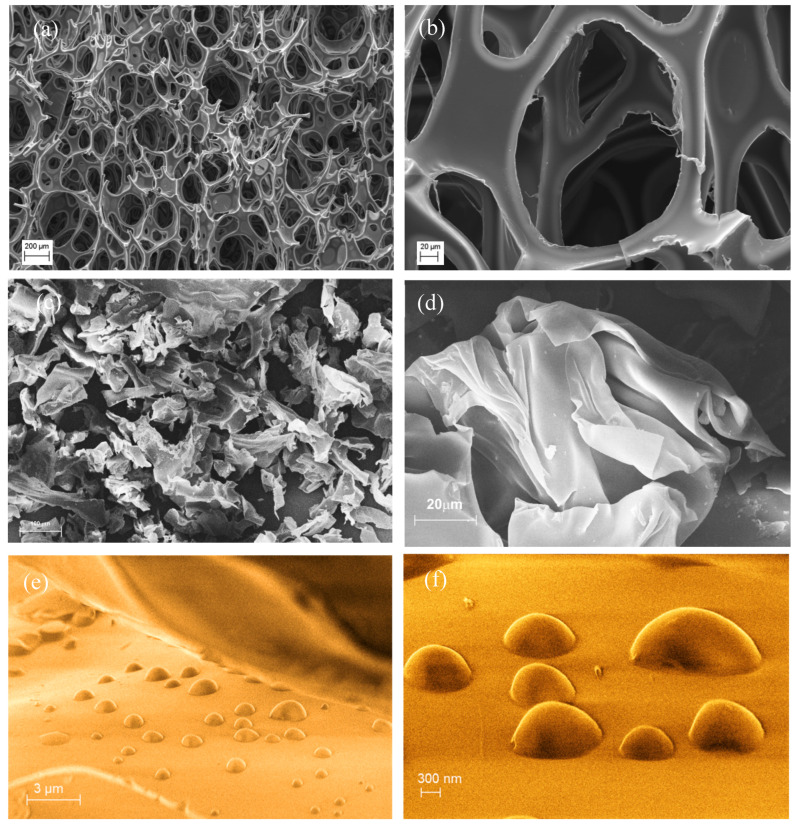
(**a**,**b**) SEM images of mixed soft PU foams before blade milling at different magnifications. (**c**) Images of fragmented cell structure of mixed soft BMG-PU powder with intermediate size fraction (−250 µm–1 mm), and (**d**) a magnification of the typical flake structure of PU fragment generated by mechanical treatment. (**e**,**f**) Different magnifications of oil droplets on the surface of BMG-PU powder after oil uptake.

SEM analysis performed on both the samples (Figure 6) confirmed the collapse and compression of the typical PU foam structure (Figure 6a,b and Figure S4), with the formation of small particles and fragments of irregular shapes, highlighting a more disordered spatial arrangement, such as multiple folding of the polymer sheets (see Figure 6c). These structures featured as planar sheets or thin membranes and, for the most part, were shaped into soft flakes similar to those displayed in Figure 6d.

**Figure 7:**
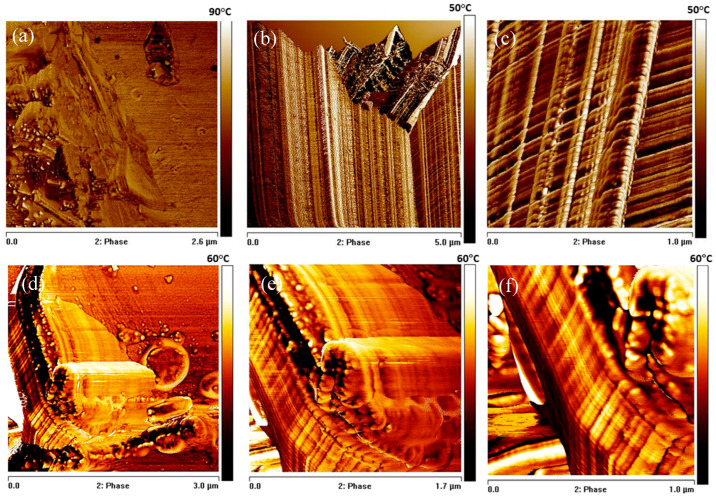
(**a**) AFM phase images of mixed soft PU foams, and the (**b**,**c**) BMG-PU highlighting the ordered microstructures recorded in the stretched and at the cut edge of the fragmented PU material. (**d**–**f**) High magnification phase images of oil sorbed on the polymer surface and within the stretched PU microstructures of BMG-PU.

The surface morphology was also investigated by AFM. The pristine soft PU foam surface (Figure 7a) exhibited the characteristic irregular and heterogeneous topography typical of microcellular PU foams. After blade milling, BMG-PUs showed a highly irregular and rough topography characterized by significant heterogeneity and fractured features (see Figure 7b,c). In line with the literature, the high contrast in the phase images revealed variations in local stiffness and adhesion arising from the presence of the soft and hard domains or urea/urethane crystallites [38,39,40]. In addition, the phase images clearly show extensive fragmented portions with well-ordered and aligned structures probably determined by the mechanical action of the blade milling process. As clearly displayed in Figure 7c, these ordered regions consist of parallel, well-aligned microstructures, perpendicularly interconnected with each other by regularly spaced and variably folded segments, composed primarily of hard domains within a softer matrix. In addition, in proximity to the cutting areas, localized deformations and rearrangements were observed. As shown in Figure 7b, blade-milling gave rise to V-shaped discontinuities and fragmentation of the surface. In some regions, the regular array of lamellae was partly preserved, whereas in others, it appeared almost completely disrupted, producing loose or protruding chain fragments and irregular domains. Small displaced fragments, attributed to chain segments exposed by mechanical scission, appeared to undergo partial 3D reorganization at the surface.

Thus, in line with the NMR analysis, the AFM observations suggest that the mechanical treatment induces considerable physical modification and chemical surface reorganization without altering the fundamental chemical structure of the PU network.

**Figure 8:**
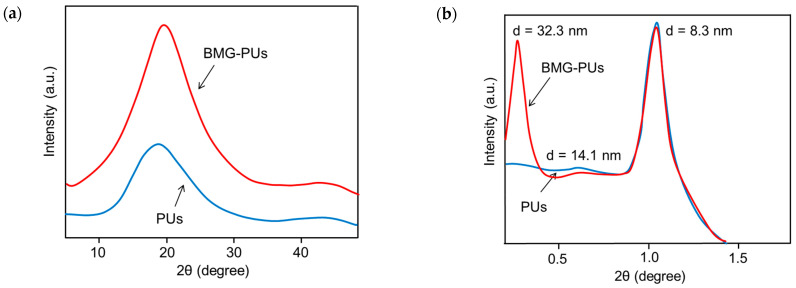
(**a**) WAXD and (**b**) SAXS profiles for mixed soft PU foams and BMG-PU powder.

To gain further insights into the structural changes induced by the milling process, mixed soft PU foams and BMG-PU powder were analyzed by X-ray diffraction (XRD) analysis. Wide-angle diffraction (WAXD) patterns of both the samples (Figure 8a) exhibited a broad peak centered at 19° 2θ and a smaller broad peak at 44º 2θ, which were due to the characteristic amorphous regions of the PU [41,42,43]. At the same time, the small-angle X-ray scattering (SAXS) analysis exhibited peaks at <2° 2θ characteristic of the typical long-range order and microphase separation of soft PU [44,45]. However, compared to the foams, the diffraction pattern of the corresponding powder (Figure 8b) revealed the appearance of a new scattering peak at a very low angle (≈0.27° 2θ), probably due to large-scale aggregates of rigid domains originating from the re-organization of hard-segment domains upon mechanical shear and compression. This is in line with the literature, where an intense signal close to the beam stop is associated with the formation of large, micron-sized aggregates of hard segments that form due to microphase separation [46].

**Figure 9:**
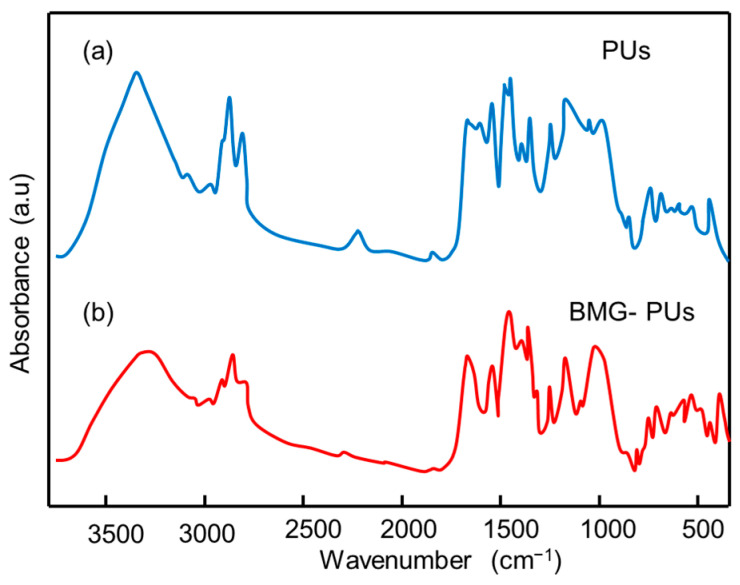
Comparison of FTIR spectra of (**a**) mixed soft PU foams, and (**b**) the corresponding BMG-PUs.

FTIR analysis was performed on mixed soft PU foams and BMG-PUs (Figure 9a,b) to investigate structural or functional micro arrangements and the potential formation of new bonds resulting from mechanochemical activation. In line with NMR, the FTIR spectrum of PU foam confirmed the characteristic structure of MDI-PPO-based PU foam [47]. In contrast, the spectrum of the BMG-PU displayed a different pattern, characterized by an increased intensity in the vibrational bands of the -CH_2_ and -CH groups (hydrocarbon chains) in the range of 2851–2972 cm^−1^, and in the –OH stretching bands at approximately 3335–3450 cm^−1^, suggesting a higher concentration of hydroxyl groups, either free or associated with polyols. Notably, changes in the C=O band associated with urethane linkages (~1725 cm^−1^), along with the disappearance of the –NCO stretching band at 2278 cm^−1^, suggest the formation of new urethane bonds via the reaction of isocyanate groups with hydroxyl groups from polyols [48]. This was further supported by the increase in the bands at 1538 cm^−1^ and 1227 cm^−1^, corresponding to N–H bending (δ(N–H)) and C–N stretching vibrations, respectively, as well as to the increase in the band at 1313 cm^−1^, attributed to a combination of N–H bending (δ(N–H)), C–N stretching (ν(C–N)), and C–H bending (β(C–H)).

**Figure 10:**
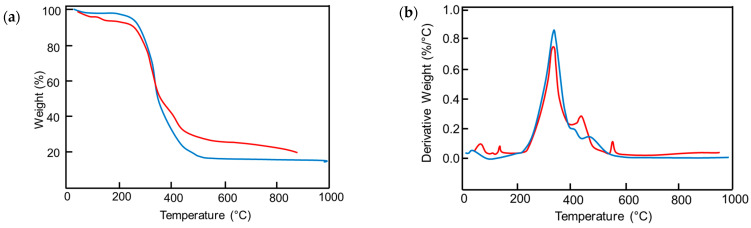
(**a**) TGA and (**b**) DTG of mixed soft PU foams (blue curves) and BMG-PUs (red curves).

Finally, TGA and DTG (Figure 10) were performed to compare the thermal behavior of soft PU foams and BMG-PUs and evaluate structural changes induced by grinding. The thermal degradation of PU foams typically occurs between 220 °C and 600° with three distinct weight loss steps corresponding to the progressive breakdown of different polymer segments and breakage of various chemical bonds (Figure 10a). Weight losses observed below 220 °C are typically attributed to the desorption of water molecules adsorbed onto the polymer surface. Based on the literature [49,50,51], the main thermal degradation step, occurring between 300 °C and 350 °C, is attributed to the decomposition of urethane bonds, while the subsequent two steps occurring at approximately 400 °C and 480 °C are due to the decomposition of ether and ester bonds, respectively. In our case, the first degradation step was responsible for ca. 70% of the total weight loss, while the other two steps contributed about 28%. Compared with PU foams, the DTG profile of BMG-PU (Figure 10b) showed an overall similarity with a difference in the distribution of weight losses. The degradation associated with the breakage of the urethane bond was around 59% of the total weight loss, and the breakdown of ether and ester bonds represented approximately 37%. This reveals that despite the formation of additional urethane linkages, through the reaction of isocyanate with hydroxyl functional groups, there was also a decrease in the urethane/ester ratio, from 2.5 to 1.6, that can be ascribed to the preferential degradation of the polyol component (see Table S4), resulting in shorter but more rigid chain segments. The relative increase in polyols is also supported by the increased weight loss observed at around 550 °C, as well as by the appearance of minor peaks below 200 °C. These low-temperature losses suggest structural modifications, i.e., indicating the presence of different types of hydrogen bonding and van der Waals interactions between water molecules and the BMG-PU.

Structural changes induced by blade milling were further evidenced by the differences observed in the DTA curves (Figure S5). Along with the two main exothermic process occurring at 300–400 °C and in the temperature range of 450–600 °C, DSC analysis showed a significant difference in the melt temperature (Tm), which was more marked for PU foams than BMG-PU, as clearly evidenced by the sharp endothermic peak at 120–130 °C (see Figure S5a,b). This supports the findings on the microstructural composition of the PU foams after grinding, which is mostly characterized by separated soft polyol components and aggregates of hard domains.

### 3.5. Oil Sorption Mechanism of PU Foam and BMG-PU Powder

In light of these results, we observed complementary oil sorption mechanisms for PU foams and their BMG-PUs counterparts. It is evident that blade milling of PU foams leads to a significant reduction in oil sorption capacity. This decrease is proportional to the extent of grinding, with the finest particle fraction (5 μm–250 μm) showing the lowest sorption capacity.

For pristine mixed soft PU foams, the high oil sorption capacity (20–30 g/g) was associated with their structural and surface features, characterized by 3D open cells, high surface area, and well interconnected pores, which facilitate capillary-driven oil uptake. As a consequence, the decrease after mechanical treatment is basically due to the rupture of this 3D porous cell structure and the concurrent reduction in surface area. In contrast, the mechanical treatment has a negligible effect on the oil sorption capacity of rigid PU foams, which show similar values (about 5 g/g) before and after grinding. This behavior is in line with the literature and can be attributed to their closed-cell structure, lower porosity, and higher density, all of which hinder oil penetration and limit the internal volume available for oil uptake.

In the case of soft BMG-PUs with variable oil sorption capacity, the key factors influencing oil uptake are related to surface chemical characteristics and their structural features, particularly involving the distribution and length of hard and soft segmented domains. As highlighted by FTIR and thermal analysis, blade milling induces an increase in the amount of the polyol (PPO) component, and consequently, a higher concentration of free hydroxyl groups. Simultaneously, hard (urethane) segments undergo fragmentations in shorter structures and tend to aggregate in larger rigid domains (structural and AFM analysis). It is also noteworthy that the energy required for ductile rupture in PU foams exceeds the intrinsic bond fracture energy. This discrepancy is due to additional energy-dissipating mechanisms such as plastic deformation occurring during fracture. Therefore, grinding causes multiscale morphological and chemico-physical surface changes, including the rupture of foam cells and plastic deformation at the mascroscale, as well as modification to the degree of phase separation with a microstructural rearrangement of segmented rigid domains at the microscale. In this regard, AFM phase images of BMG-PU (see Figure 7b,c) clearly showed the alignment of hard segments parallel to the direction of mechanical stress, along with the formation of perpendicular, interlocking hard domain structures. These considerations are in line with SAXS, which reveals the formation of large aggregates resulting from the packing of hard domains within the soft matrix. Taking into account these considerations, we speculated that, upon mechanical pulverization, the PU foams dissociate into smaller and more regular fragments, whereas hard segments tend to aggregate into larger structures within the soft matrix. This results in aggregated hard segments with greater separation, contributing to altered oil sorption behavior.

As a result, the disruption of PU foams and the associated microstructural rearrangement of segmented domains, characterized by shortened hard segments, reduced hydrophilic urethane content, and increased polyol concentration, play a role in the oil sorption capacity of the different soft BMG-PU powders, especially for those with intermediate particle dimensions (−250 μm–1 mm). The chemico-physical surface and morphological reorganization resulted in densely packed oleophilic domains, which in turn influences the oil uptake, as shown in AFM phase images (Figure 7d–f) and SEM micrographs, where oil droplets visibly adhered to the surface and filled oleophilic regions through a coalescence mechanism, similar to a “sandwich effect” (Figure 6e,f and Figure S6).

## 4. Conclusions

In this work, we studied the oil sorption capacity of recycled PU foam wastes, both rigid and soft, and the corresponding blade-milled ground (BMG)-PU powders obtained by blade-milling treatment. The effect on the oil sorption of BMG-PUs was also investigated with respect to the morphological, microstructural, and chemical modifications induced by mechanical action. The grinding process gave rise to two particle fractions of BMG-PUs with different sizes, ranging from 250 μm to 1 mm and 5 μm to 250 μm, as clearly highlighted by laser diffraction and optical microscopy. The oil sorption measurements revealed that pristine soft PU foams and BMG-PUs with intermediate particle size (250 μm–1 mm) exhibited the highest oil uptake (20–30 g/g), whereas those with the finest particle sizes (5 μm–250 μm) showed a lower capacity (3–7 g/g). In contrast, rigid PU foams showed the lowest oil sorption values (~5 g/g) with negligible differences between the foams and the BMG-PU powder. By the evaluation of the structural and morphological changes induced by the grinding process, the decrease in oil sorption after mechanical treatment, especially for smaller particle fractions, is attributed to the rupture of the typically 3D porous structures and interconnected cells of PU foams, which are responsible for effective oil uptake by acting as a “cage” for the oil. The collapse of these structures and morphological features was demonstrated by SEM and AFM characterizations. The evaluation of the structural and chemico-physical changes induced by blade milling was performed on the most absorbent PU foam, i.e., mixed soft PUs, before and after grinding, by combining X-ray diffraction with spectroscopic FTIR analysis and the study of thermal behavior (TGA/DTG, DTA). Interestingly, we found that the mechanical treatment determined a rearrangement at the microscale of the segmented domains of the polymer chains characterized by shortened and aggregated hard segments, reduced hydrophilic urethane content, and increased polyol concentration. These modifications enable the formation of more densely packed oleophilic domains, affecting the oil uptake, where oil droplets adhere to the surface and fill oleophilic regions. Therefore, in a different way from the pristine PU foams, the BMG-PU powder presents a different sorption mechanism, where the resulting fragments of the disrupted foam structure, characterized by different segmented domain arrangements with aggregates of hard domains within the soft phase, capture small oil droplets through a coalescence mechanism similar to a “sandwich effect”. The subsequent aggregated oil/powder leads to an increase in the total volume and stabilization of oil within the polymer sorbent.

Although there was reduced performance compared to pristine PU foams, the absorbing BMG-PU powders, especially those with intermediate dimensions originating from soft PU foams, can represent an alternative solution for oil sorption and eventually for oil spill cleanup, making the management of PU foam waste materials simultaneously more sustainable, more versatile, and less expensive.

## Data Availability

The original contributions presented in this study are included in the article/Supplementary Material. Further inquiries can be directed to the corresponding authors.

## Supplementary Materials

The following supporting information can be downloaded at: https://www.mdpi.com/article/10.3390/ma19010166/s1. Figure S1. Comparison between particle size distribution in water (red) and in air (green) of BMG-PU wastes; Figure S2. Light transmission measured on 20540 particles of BMG-PU waste material. The intensity mean was smoothed over 11 points. Transmission values were plotted against arbitrary light transmission units (a.l.t.u.), where 0 corresponds to the complete extinction of light and 260 to the complete transmission; Figure S3. Oil sorption capacity of BMG-PU powder with particle size ranging from 1 mm to 250 μm (left side) and 250 μm to 5 μm (right side); Figure S4. SEM image of mixed soft PU foam sample before the mechanical treatment; Figure S5. (a,b) DTA of mixed soft PU foams before and after blade milling (BMG-PUs), respectively; Figure S6. Surface characteristic of BMG-PU and contact angle measured from SEM image; Table S1. Results of grinding yields for BMG-PUs with two-size particle fractions; Table S2. Comparison of oil sorption capacity for different type of PU foams reported in the literature from 2013 to 2025; Table S3. Main chemico-physical properties of mixed soft BMG-PUs produced by mechanical treatment of recycled flexible PU foams; Table S4. Ratios of Urethane and Ester observed in PU foams and BMG-PUs.
