# Supplementary material for: Oil Sorption Capacity of Recycled Polyurethane Foams and Their Mechanically Milled Powders
Source: Materials (Basel). 2026 Jan 2;19(1):166. doi: 10.3390/ma19010166 (PMC12786837; doi:10.3390/ma19010166)
Supplement: Supplementary file 1 [file materials-19-00166-s001.zip › materials-4058065-supplementary.pdf]

SUPPLEMENTARY INFORMATION

Oil Sorption Capacity of Recycled Polyurethane Foams and Their Mechanically Milled Powders

Pierluigi Cossari [1,*], Daniela Caschera [2,*] and Paolo Plescia [3]

1 Department of Physics, Institute of Nanotechnology CNR-NANOTEC, Sapienza University, 00185 Roma, Italy
2 Institute for the Study of Nanostructured Materials, Lab Surface, ISMN-CNR, Strada Provinciale 35d/9, Montelibretti, 00010 Roma, Italy
3 Institute of Environmental Geology and Geoengineering, CNR-IGAG, Strada Provinciale 35d/9, Montelibretti, 00010 Roma, Italy; ilplescia@gmail.com
* Correspondence: pierluigi.cossari@cnr.it (P.C.); daniela.caschera@cnr.it (D.C.)

Table S1. Results of grinding yields for BMG-PUs with two-size particle fractions.

Type of PU foams	Yields	
	250 µm – 1 mm (%)	< 250 µm (%)
**Soft PU _RT**	*85-90*	*10-15*
**Soft PU –N_2**	*55-60*	*40-45*
**Rigid PU – RT**	*65-80*	*20-35*

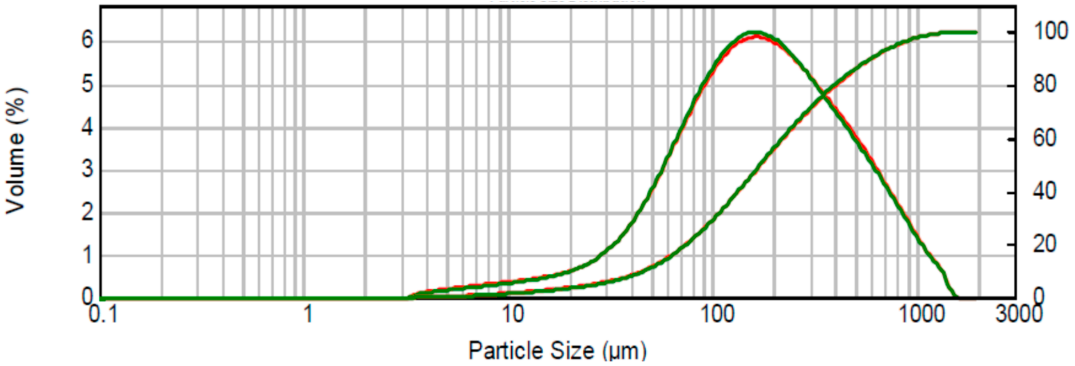

Figure S1. Comparison between particle size distribution in water (red) and in air (green) of BMG-PU wastes.

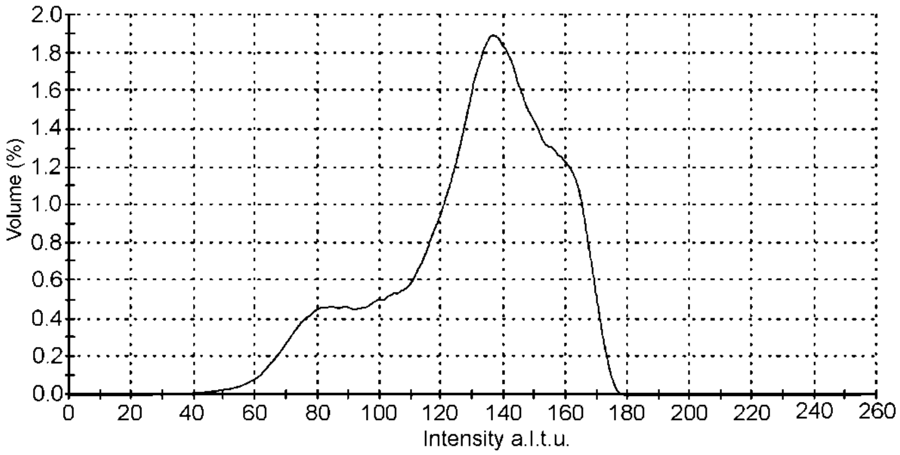

Figure S2. Light transmission measured on 20540 particles of BMG-PU waste material. The intensity mean was smoothed over 11 points. Transmission values were plotted against arbitrary light transmission units (a.l.t.u.), where 0 corresponds to the complete extinction of light and 260 to the complete transmission

Table S2. Comparison of oil sorption capacity for different type of PU foams reported in the literature from 2013 to 2025.

References	PU and PU based materials	Type/ shape	Dimensions (cm-µm)	Oil sorption capacity (g/g)						
[18] A. De Folli et al. 2023	FoamFlex200 d=30 kg/m^3	Flexible Foams	Thickness = 2.5 cm		Crude oil C-1564<br>23.2 ± 1.4		Diesel<br>18.8 ± 1.2		Kerosene<br>8.8 ± 0.6	
[16] V. Santucci et al. 2021					Diesel fuel, Quaser Q8		Motor Oil 10w40		Motor Oil 20w50	
	Recycled PU from refrigerator panels (fridge)	Powder	d < 0.71 mm; (d=129.65 kg/m^3)		4.51 ± 0.64		5.11 ± 0.50		7.02 ± 1.49	
			0.71< d < 5; (d= 47.57 kg/m^3)		4.17 ± 0.31		7.36 ± 0.50		6.41 ± 2.41	
			d > 0.71; (d= 42.00 kg/m^3)		7.07 ± 0.23		7.72 ± 0.80		10.30 ± 0.89	
			2 < d < 4 sepiolite ; (d= 440 kg/m^3)		3.26 ± 0.24		4.60 ± 0.30		5.27 ± 0.11	
			d < 1 OKO-PUR; (d= 127.5 kg/m^3)		1.30 ± 0.01		1.34 ± 0.14		1.64 ± 0.06	
[24] Ma et al. 2021	Lignin based PU foam with CNTs	Foam					Crude oil<br>c.a. 6.35 g/g			
[25] Li et al. 2025	Lignin based PU foam liquified lignin polyols (LLP) + Hexamethil diisocianate (HDI)	Foam		Hexane<br>4	Hexadecan<br>5	Octan<br>7.7	Toluene<br>10.8	Benzene<br>9	THF<br>12	CH_2Cl_2<br>20
[26] P. Calcagnile et al. 2012	PU foam –Fe nanoparticles	Foam	2.0 × 1.5 × 0.2 cm3 (length, width, thickness)				Mineral oil<br>12			
[23] Tomon et al. 2024	Control PU Foam	Foam:Polyol + pMDI		Water<br>6-7	Seawater<br>5	Vegetable oil<br>13	Engine oil<br>2.6	Used engine oil<br>2.4	Bunker fuel<br>15	
	Naturally superolephilic foam (NSF)	Polyols coconut oil + pMDI		1.5	1	16	15	17.6	23.5	
[27] H. Li et al.2013	Control PU Foam	Foam			Water<br>14.1		Diesel<br>39.7 ??		Kerosene<br>39.9??	
	Lauryl methacrylate foam (LMF)	Foam			7.05		46.98		41,42	
[21] Al-Khalaf, 2022	Flex-PU (FPU)	Soft foam	n.a		Water		Water		Water	
	FPU-0				18		29 ??		27 ??	
	FPU-A1				7.7		37		36	
	FPU-A2				7.6		42		39	
	FPU-A3				7.58		45		41	
[22] Liu et al. 2014	PUR control	63 PU	n.a		Toluene<br>6-16		Gasoline<br>6-12		Diesel fuel<br>6-11	
	HPF:(HDS-*g*-HTPB) + MDI	57PU								
		84PU			27		18		18	
		133PU			25		14		14	
					23		8		8	

Table S3. Main chemico-physical properties of mixed soft BMG-PUs produced by mechanical treatment of recycled flexible PU foams.

Properties	Values
Density	0.16 g/cm^3
Color	Light brown
Heat of combustion	25 MJ/kg
Melting Temperature	220 °C
Water content	4 % w/w
Ash (600 °C)	27 %

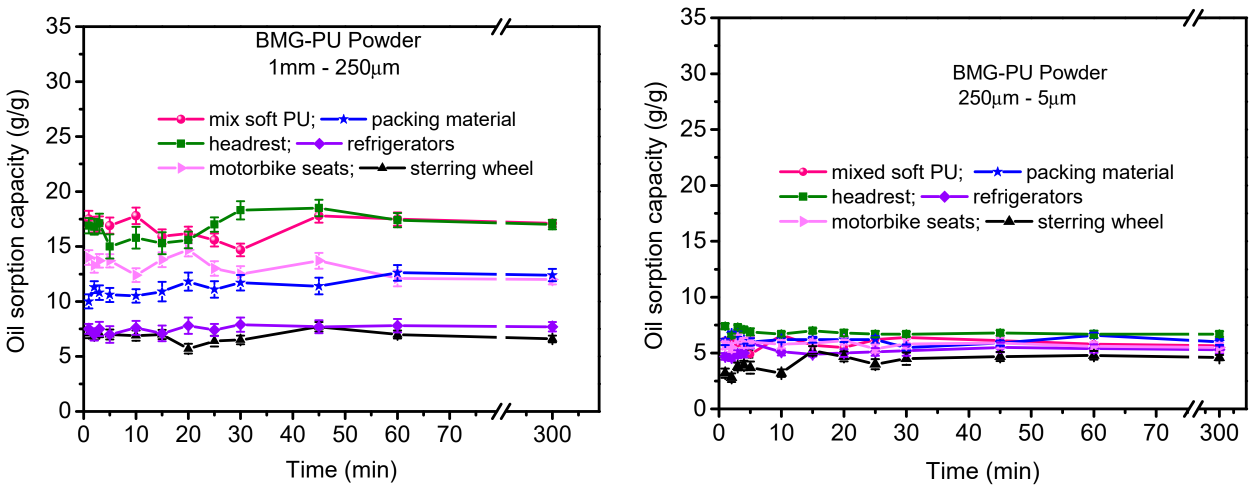

Figure S3. Oil sorption capacity of BMG-PU powder with particle size ranging from 1mm to 250 µm (left side) and 250 µm to 5 µm (right side).

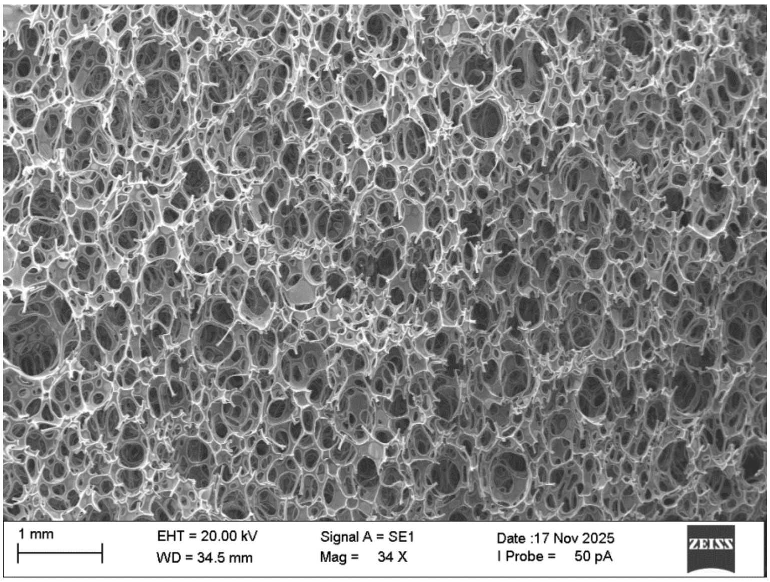

Figure S4. SEM image of mixed soft PU foam sample before the mechanical treatment.

Table S4. Ratios of Urethane and Ester observed in PU foams and BMG-PUs.

**Materials**	**urethane %**	**ester %**	**urethane/ester ratio**
Mix soft PU foam	71	28	2.5
BMG-PU powder	59	39	1.5

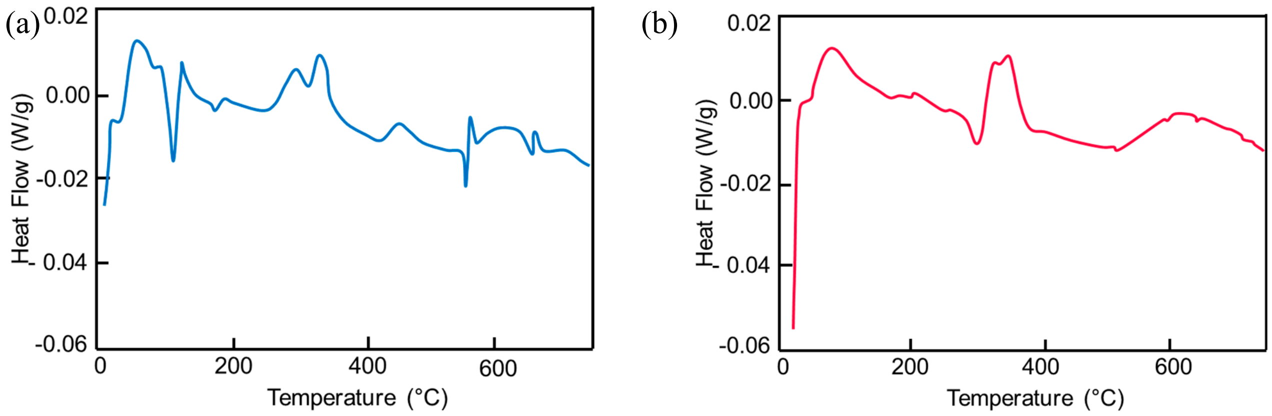

Figure S5. (a,b) DTA of mixed soft PU foams before and after blade milling (BMG-PUs), respectively.

Supplementary Figure S5 shows the DTA of mixed soft PU foams and BMG-PUs, respectively. The small peaks in the thermograms are baseline noise.

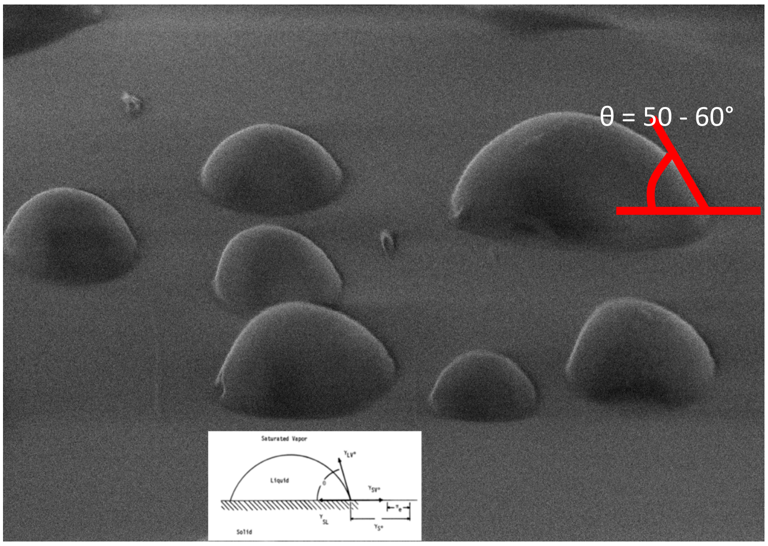

Figure S6. Surface characteristic of BMG-PU and contact angle measured from SEM image.
